# Differential Regulation of Innate Lymphoid Cells in Human and Murine Oral Squamous Cell Carcinoma

**DOI:** 10.3390/ijms24021627

**Published:** 2023-01-13

**Authors:** Sofia Ali Syed, Muhammad Asif Qureshi, Saeed Khan, Rajesh Kumar, Iqbal A. Muhammad Khyani, Bilal Ahmed Khan, Jawad Safdar

**Affiliations:** 1 Department of Oral Pathology, Dr. Ishrat-ul-Ebad Khan Institute of Oral Health Sciences, Dow University of Health Sciences, Karachi 75300, Sindh, Pakistan; 2 Department of Pathology, Dow International Medical College, Dow University of Health Sciences, Karachi 75300, Sindh, Pakistan; 3 Department of Otorhinolaryngology, Head and Neck Surgery, Dow Medical College, Dow University of Health Sciences, Karachi 74200, Sindh, Pakistan; 4 Department of ENT, Head and Neck Surgery, Shaheed Mohtarma Benazir Bhutto Medical College, Lyari General Hospital, Karachi 74200, Sindh, Pakistan; 5 Oral & Maxillofacial Surgery, Dow Dental College, Dow University of Health Sciences, Karachi 74200, Sindh, Pakistan

**Keywords:** cytokine, human, innate lymphoid cells, murine, oral squamous cell carcinoma, tumor burden

## Abstract

Oral squamous cell carcinomas (OSCC) remain a major healthcare burden in Asian countries. In Pakistan alone, it is the most common cancer in males and second only to breast cancer in females. Alarmingly, treatment options for OSCC remain limited. With this context, investigations made to explore the inflammatory *milieu* of OSCC become highly relevant, with the hope of practicing immunotherapeutic approaches to address this highly prevalent tumor. We investigated the newly identified innate lymphoid cells (ILCs) and associated cytokines in well-defined human oral squamous cell carcinoma (OSCC) as well as in a 7,12-dimethylbenz[a]anthracene (DMBA)-induced murine model of OSCC using flow cytometry and quantitative real-time polymerase chain reaction (qPCR). We further went on to explore molecular circuitry involved in OSCC by developing a murine model of OSCC and using an α-Thy1 antibody to inhibit ILCs. Amongst the ILCs that we found in human OSCC, ILC3 (23%) was the most abundant, followed by ILC2 (17%) and ILC1 (1%). Mice were divided into four groups: DMBA (n = 33), DMBA+antibody (Ab) (n = 30), acetone (n = 5), and control (n = 5). In murine OSCC tissues, ILC1 and ILC3 were down-infiltrated, while ILC2 remained unchanged compared to controls. Interestingly, compared to the controls (DMBA group), mice treated with the α-Thy1 antibody showed fewer numbers of large tumors, and a larger percentage of these mice were tumor-free at this study’s end point. We present novel data on the differential expansion/downsizing of ILCs in OSCC, which provides a pivotal basis to dive deeper into molecular circuitry and the OSCC tumor niche to devise novel diagnostic, therapeutic, and prognostic strategies to prevent/treat oral cancers.

## 1. Introduction

Oral squamous cell carcinoma (OSCC) is the most frequent malignancy in Pakistani males and the second most common malignancy in females [1]. Worldwide, the 5-year survival rate is less than 50%, indicating that OSCC is a major health care burden [2]. OSCC propagates in a highly inflammatory environment composed of cancer-associated fibroblasts (CAFs), immune cells including T cells, myeloid-derived suppressor cells (MDSCs), and macrophages, which have tumor-promoting as well as tumor-protective activities [3]. Moreover, various inflammatory pathways, including nuclear factor kappa B (NF-_K_B), nuclear signal transducers and activators of transcription 3 (STAT-3), mitogen-activated protein kinase (MAPK), activator protein1 (AP1), and cyclooxygenase (COX)-2, have been reported to play crucial roles in OSCC [4]. This is further orchestrated by the secretion of various inflammatory players including transforming growth factor-β (TGF-β), tumor necrosis factor-α (TNF-α), interleukin (IL)-1, IL-6, and IL-8, all of which promote a tumor-supportive environment for OSCC progression [5].

Innate lymphoid cells (ILCs) are a new category of lymphocytes that are found to be expanded in various tumor types, including OSCC [6,7]. ILCs are lineage negative (LIN-) cells that lack receptors which are generally found on T and B cells. ILCs are broadly classified into three groups: ILC1, ILC2, and ILC3, which mirror the functional characteristics of adaptive T-helper cell subsets including T_H_1, T_H_2, and T_H_17, respectively. Group 1 ILCs consist of natural killer cells (NK) and ILC1. ILC1s express T-bet transcription factor and produce cytokines such as interferon (IFN)-γ and tumor necrosis factor (TNF)-α that are perilous against viral infections and cancers. ILC2s express the transcription factor GATA-binding protein 3 (GATA3) and secrete interleukin (IL)-4, IL-5, and IL-13. Group 3 ILCs produce IL-17 and IL-22 and depend on the RAR-related orphan receptor gamma t (RORγt) transcription factor. Group 3 ILCs are further categorized into natural cytotoxicity receptor (NCR)^+^ and NCR^−^ ILC3s, as well as lymphoid tissue inducer (LTi) cells [8]. With mounting evidence, it is now known that ILCs react to environmental signals, release cytokines, and alter their type and function in inflammation and cancers [6]. While the contribution of innate and adaptive immunity as well as cytokines in OSCC is well-studied [9], data related to the inflammatory infiltrate in OSCC is scarce in Pakistan, with only a few studies reported in the literature [10,11]. It is therefore highly relevant to investigate ILCs, new players in the tumor-immune niche that may play dual roles in the OSCC tumor environment.

In this study, we investigated ILCs infiltration and expression of associated cytokines in well-defined human samples of OSCC. We further investigated the role of ILCs using a murine model of OSCC. Our findings highlight ILCs as important players in OSCC pathogenesis, thus opening avenues for identifying molecular players of diagnostic, therapeutic, and prognostic significance.

## 2. Results

### 2.1. Clinicopathologic Characteristics of Human Oral Squamous Cell Carcinoma

**Figure 1:**
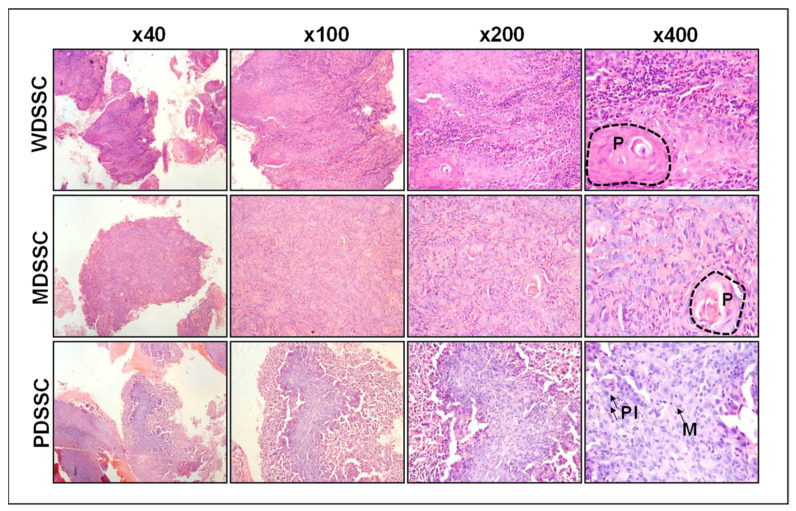
Representative images of hematoxylin and eosin (H & E) stained oral squamous cell carcinoma at x40, x100, x200, and x400 magnification. Well-differentiated squamous cell carcinoma (WDSCC) and moderately differentiated squamous cell carcinoma (MDSCC) showing large and small keratin pearls (P), respectively. Poorly differentiated squamous cell carcinoma (PDSCC) showing mitosis (M) and pleomorphism (Pl).

Of the 52 OSCC patients that were recruited for this study, 42 (80.7%) were males and 10 (19.2%) were females. The mean-age of our study recruits was 46 years. The most common site was buccal mucosa (53.2%), followed by the tongue (10%). The majority of the patients (80.8%) were tobacco users. Histopathologic analysis showed that 38 (73.07%) cases were moderately differentiated, 9 (17.3%) were well differentiated, and 5 (9.6%) were poorly differentiated (Figure 1). Of the 52 patients, 29 had undergone surgical excisions and 23 had undergone punch biopsies. Of 29 patients, 13 (44.8%) showed tumor (T) size in the T2 category, 11 (37.9%) were in the T4 category, and 14 (48.3%) showed lymph node metastasis. In addition, 27 (93.1%) patients showed no lymphovascular invasion, 11 (37.9%) showed perineural invasion, and only 1 (3.4%) showed submandibular gland involvement, as shown in Table 1.

### 2.2. Human OSCC Microenvironment Exhibit Increased Lymphocytic Infiltrate

**Figure 2:**
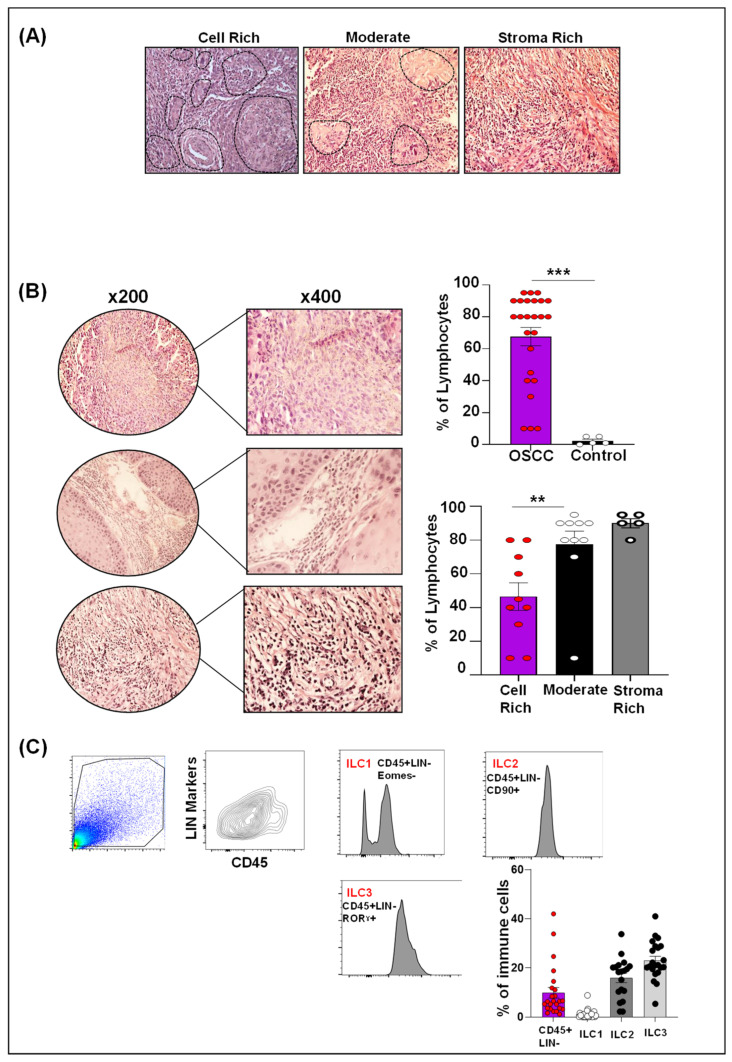
**Lymphocyte and Innate lymphoid cells (ILCs) infiltration in human oral squamous cell carcinoma (OSCC).** (**A**) Representative images of H & E-stained sections showing cell-rich, moderate, and stroma-rich areas (black dotted line showing tumor cells); (**B**) Evaluation of lymphocytes in cell-rich, moderate, and stroma-rich areas at x200 and x400 magnification. Increased lymphocytic infiltration in OSCC compared to control. Lymphocyte infiltration was highest in stroma-rich areas, followed by moderate and cell-rich areas. (**C**) Gating strategy and quantification of CD45+ Lin- cells, ILC1, ILC2, and ILC3 show increased infiltration of ILC3 (23%), followed by ILC2 (17%) and ILC1 (1%). ** *p* < 0.01, *** *p* < 0.0001.

There was a significant increase in the infiltration of lymphocytes in OSCC tissues compared to non-tumor tissue controls (*p* < 0.05). The highest infiltration was observed in stroma-rich tumors, followed by moderate tumors, and least infiltration was observed in tumor cell-rich tumor types (*p* < 0.05) (Figure 2A,B).

### 2.3. Differential ILCs Expansion in Human OSCC

In order to investigate ILC infiltration in human OSCC tissues, we investigated OSCC as well as control (non-tumor adjacent) tissues. Very few mononuclear cells were isolated from controls and therefore could not be further analyzed for ILC categorization. OSSC tissues, however, were infiltrated with a considerable number of mononuclear cells, which were further analyzed for ILCs. Amongst the three ILC subsets investigated in human OSCC tissues, the ILC3 (23%) infiltrate was most abundant, followed by the ILC2 (17%) and the ILC1 (1%) (Figure 2C). We further analyzed ILC subsets separately in various categories of clinicopathological parameters as outlined in Table 1 (Supplementary Figure S1). While we did not see any significant differences across various parameters, there were some interesting trends to comment on. For example, ILC1 infiltration was highest in OSCC originating from buccal mucosa compared to ILC2 and ILC3 infiltration, which were more commonly seen in OSCC originating from the lips and tongue. There was an upward trend in all ILCs infiltration in tobacco non-users. Importantly, an upward trend of ILCs infiltration was noticed as OSCC grade advanced, low in well-differentiated tumors and highest in poorly differentiated tumors. Lastly, OSCC with perineural invasion (PNI) exhibited higher ILC infiltration (particularly ILC2 and ILC3) compared to those without PNI. These interesting findings/trends demand detailed characterization with an increased sample size to delineate the significance of the observed trends.

### 2.4. ILC1 and ILC3 Are Deregulated in Murine Model of OSCC

**Figure 3:**
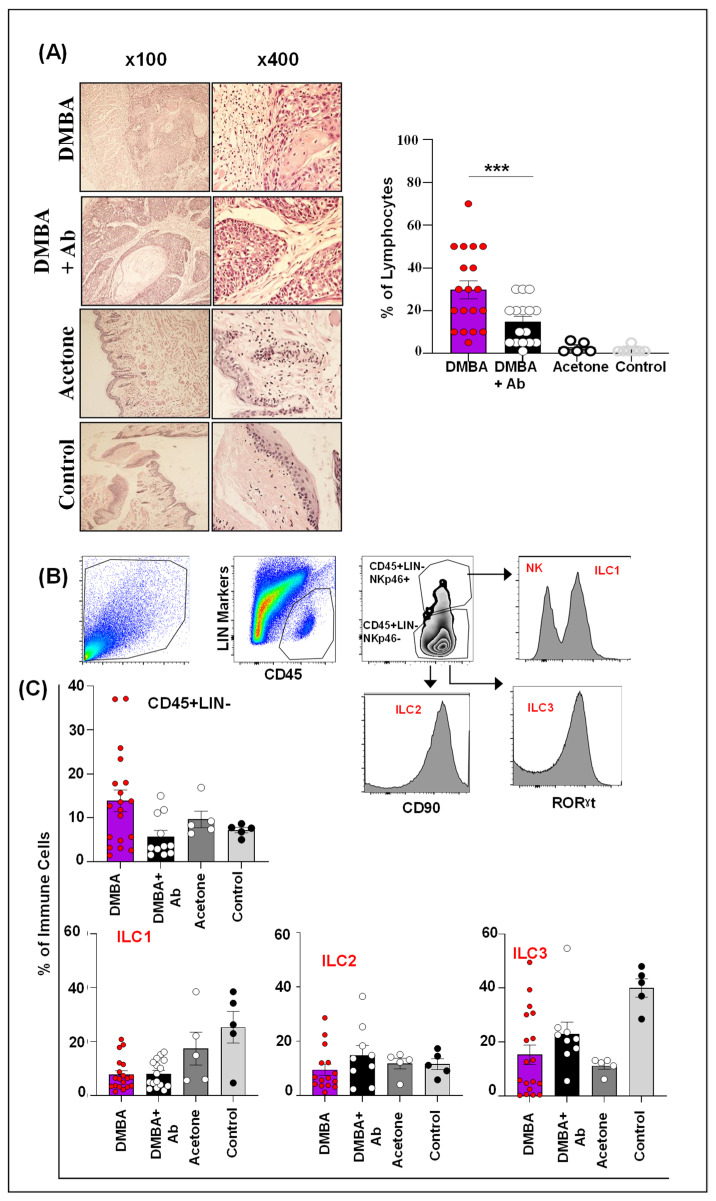
**Expression of lymphocytes and ILC population in murine OSCC.** (**A**) Representative H & E images of lymphocyte infiltration in DMBA, DMBA+Ab, acetone, and control groups at 100x and 400x magnification. Increased infiltration of lymphocytes in DMBA and DMBA+Ab group mice compared to mice in the acetone and control groups. Lymphocytic infiltration was significantly higher in DMBA group mice compared to DMBA+Ab group mice (*p* < 0.05). (**B**) Gating strategy for ILC1, ILC2, and ILC3. (**C**) Increased infiltration of CD45+LIN- cells in DMBA group mice. ILC1 and ILC3 were significantly decreased in DMBA and DMBA+Ab mice groups compared to control mice, while the ILC2 infiltrate was not significantly different amongst various mice groups. DMBA = 7,12-dimethylbenz[a]anthracene; DMBA+Ab = 7,12-dimethylbenz[a]anthracene+antibody. *** *p* < 0.001.

In the murine model of OSCC, the percentages of lymphocyte infiltration in the DMBA and DMBA+antibody (Ab) groups were high compared to the acetone and control groups. In addition, the lymphocyte infiltration was significantly increased in the DMBA group compared to the DMBA+Ab group (*p* < 0.05) (Figure 3A). Next, we investigated the ILCs population among the infiltrated lymphocyte by using the gating strategy as defined in the methodology section (Figure 3B). The highest percentage of CD45+LIN- cells were infiltrated in the DMBA group. Interestingly, ILC1 and ILC3 were significantly reduced both in DMBA and DMBA+Ab groups compared to controls, while ILC2 infiltrate was not significantly different amongst the various groups investigated (Figure 3C). Collectively, these data demonstrate differential expansion/infiltration of ILCs in the DMBA group compared to controls, which in principle equates to findings (particularly for ILC1) in human OSCC tissues.

### 2.5. ILC Inhibition Decelerates Tumorigenesis and Limits Tumor Expansion In Vivo

**Figure 4:**
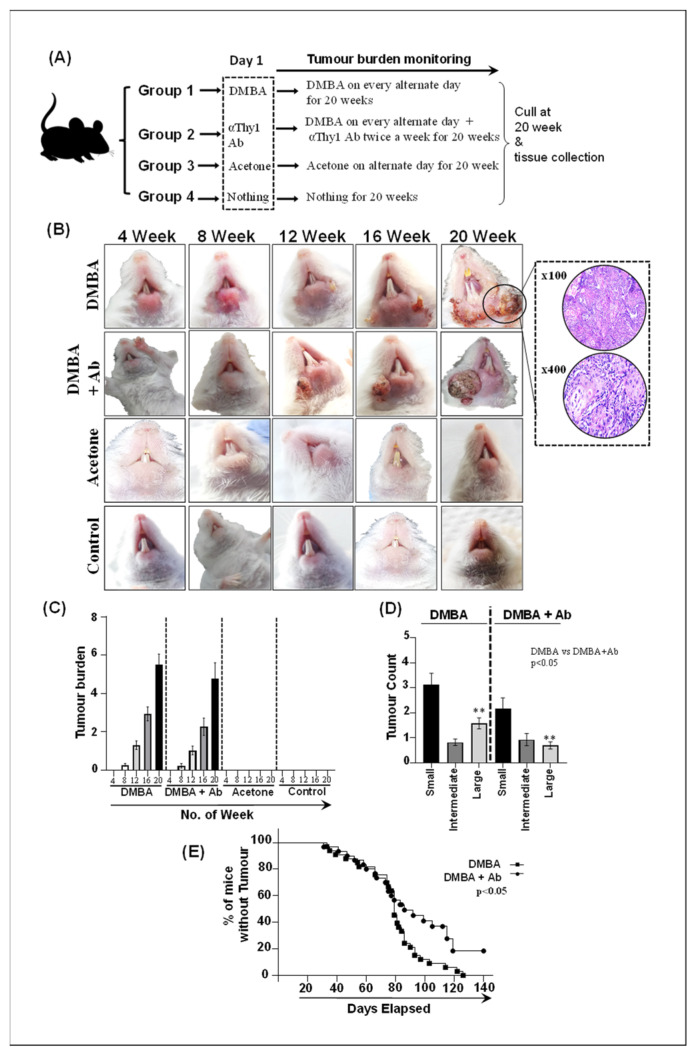
**Representative images of murine experiment showing tumor burden, tumor size, and the percentage of mice with tumors.** (**A**) Mice were divided into groups as follows: the DMBA group (n = 33), the DMBA+Ab group (n = 30), the acetone group (n = 5), and the control group (n = 5). Mice in the DMBA and DMBA+Ab groups were topically brushed with DMBA on the lower lip every other day for 20 weeks. Mice in DMBA+Ab also received intraperitoneal injections of the antiThy1 antibody (300 μL) twice a week for 20 weeks. Mice in the acetone group were brushed with acetone on the lower lip every other day for 20 weeks, while mice in the control group received nothing. (**B**) Tumor burden was monitored at 4, 8, 12, 16, and 20 weeks. Mice in the DMBA and DMBA+Ab groups developed tumors that were histopathologically confirmed as oral squamous cell carcinoma. Mice in acetone and control groups did not develop tumors. (**C**) Tumor burden was reduced in DMBA+Ab mice compared to DMBA mice at weeks 8, 12, 16, and 20; however, the difference was not significant (*p* > 0.05). (**D**) Small tumors were highest in numbers in both the DMBA and DMBA+Ab groups of mice. Large tumors in DMBA+Ab mice were significantly lower than in DMBA mice. (**E**) The Kaplan–Meier curve showed that at day 140 (20 weeks), all mice (100%) in the DMBA group developed tumors, whereas in the DMBA+Ab group, 10 (33.33%) mice were tumor-free (*p* < 0.05). ** *p* < 0.01.

To investigate the role of ILCs in OSCC, ILCs were blocked in vivo using the antiThy1 antibody in DMBA+Ab group mice, and various murine groups were compared for tumor burden, tumor count, and percentage of mice with and without tumor at the study end point (Figure 4A–E). All mice in the DMBA and DMBA+Ab groups developed tumors, while none was developed in any of the mice in the acetone and control groups (Figure 4A,B). Mice in the DMBA and DMBA+Ab groups did not show any significant differences in terms of days taken to develop the first lesion. The first lesion in the DMBA group mice was observed at day 33, while mice in the DMBA+Ab group showed a first lesion at day 31. Average days for the mice in the DMBA and DMBA+Ab groups to develop lesions were 78.54 and 75 days, respectively. The tumor burden (total tumor count per mouse) was recorded at weeks 4, 8, 12, 16, and 20. Interestingly, the tumor burden was reduced in DMBA+Ab mice compared to DMBA mice at week 8 (13.33% vs. 27.27%), week 12 (50% vs. 66.66%), week 16 (60% vs. 93.93%), and week 20 (66.66% vs. 100%); however, the difference was statistically not significant (*p* > 0.05) (Figure 4C). Tumors in the DMBA and DMBA+Ab groups of mice were divided into small (<2 mm), intermediate (>2–4 mm), and large (>4 mm) categories. Overall, small tumors were highest in numbers in both the DMBA and DMBA+Ab groups of mice. Although DMBA+Ab mice had a declining trend of small tumors compared to DMBA mice, it was not statistically significant. Importantly, large tumor counts in DMBA+Ab mice were significantly lower compared to DMBA mice, suggesting limited tumor expansion in mice treated with the anti-Thy1 antibody (Figure 4D). We further investigated the proportion of mice with and without tumors over the study’s time period. Importantly, the DMBA+Ab mice group exhibited a higher percentage of mice without tumors. The Kaplan–Meier curve analysis showed that at day 140 (the study endpoint), all mice (100%) in the DMBA group developed tumors, whereas in the DMBA+Ab group, 10 (33.33%) mice were tumor-free, and the difference was significant (*p* < 0.05) (Figure 4E). Collectively, these data indicate that ILCs blockade decelerates oral tumorigenesis and limits tumor expansion in the murine model of OSCC.

### 2.6. Expression of Cytokines in Human and Murine Oral Squamous Cell Carcinoma

**Figure 5:**
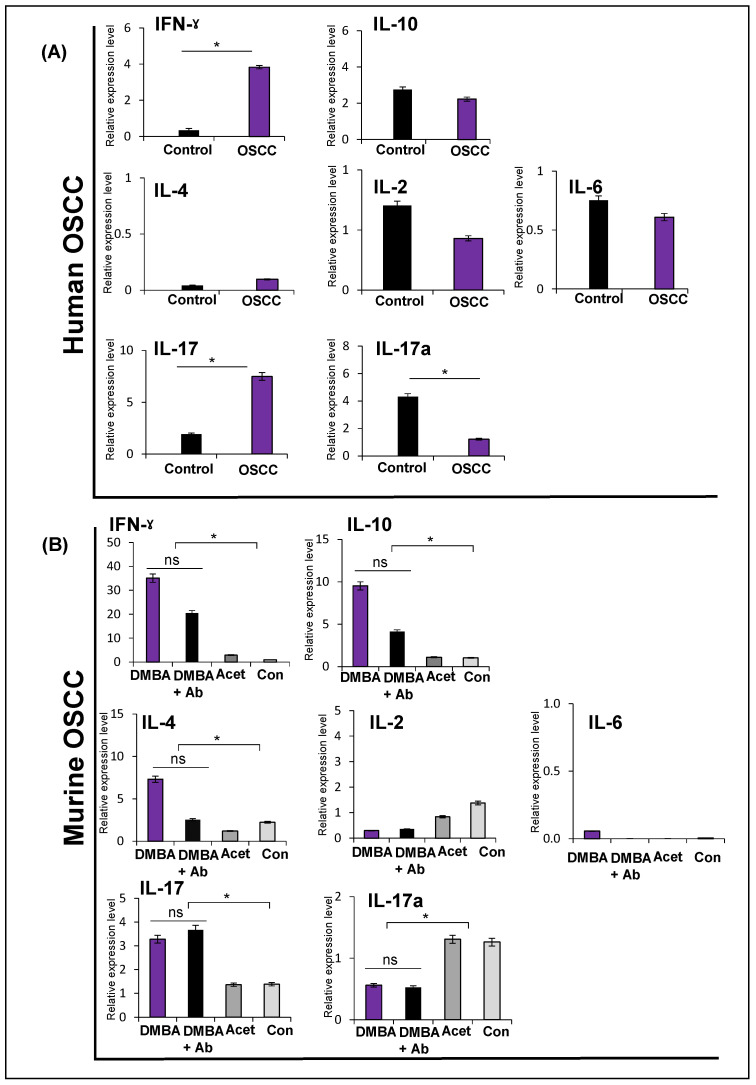
**Expression of human and murine cytokines in oral squamous cell carcinoma.** (**A**) In human OSCC (n = 15), IFNγ and IL-17 showed significantly higher expression in OSCC than non-tumor adjacent tissue controls (n = 5) whereas IL-17a was low compared to non-tumor tissue controls. IL-10, IL-4, IL-2, and IL-6 expression were not different in OSCC compared to controls. (**B**) In murine OSCC, expression of IFNγ, IL-10, and IL-4 was significantly higher in the DMBA and DMBA+Ab groups compared to the controls. However, IL-2 and IL-6 were not different among groups. IL-17 was also high in both DMBA and DMBA+Ab groups, while IL-17a was low in both DMBA and DMBA+Ab groups. (* = *p < 0.05, ns = not significant).* SEM are represented as error bars. IL—interleukin; Acet—acetone; and Con—control.

We also investigated the expression of a few selected cytokines, including representatives of each ILC subset. In human OSCC, IFNγ was significantly higher compared to controls (*p* < 0.05). IL-10 expression was not different in OSCC and controls. IL-4, IL-2, and IL-6 were not different from controls (*p* > 0.05). IL-17 was significantly higher, while IL-17a was significantly lower compared to controls (*p* < 0.05) (Figure 5A).

In murine OSCC, IFNγ, IL-4, and IL-10 showed significantly high expression in the DMBA group (*p* < 0.05), while IL-2 and IL-6 were not different amongst groups (*p* > 0.05). IL-17 was high in the DMBA and DMBA+Ab groups, while IL-17a was down in the DMBA and DMBA+Ab groups (*p* < 0.05) (same finding as in human OSCC) (Figure 5B). If we look at these data further, it is evident that expression of IFNγ, IL-4, IL-10, and IL-17 also showed a trend of upregulation in the DMBA+Ab group. However, differences in these expression levels amongst the DMBA and DMBA+Ab groups were not statistically significant. Taken together, selected cytokines show deregulated expression patterns in both the DMBA and DMBA+Ab groups compared to the controls. While the differences in the expression of these cytokines were significantly different between OSCC (including DMBA and DMBA+Ab) and controls, it was not statistically different amongst the DMBA and DMBA+Ab groups (limiting us to further analyze these groups for the ILCs sub-category analyses).

## 3. Discussion

OSCC has increased mortality and morbidity with limited therapeutic options, including surgery with adjuvant chemotherapy and/or radiotherapy [12]. Previous research focused on targeting genetic mutations in tumor cells; however, the recent studies unraveling the cross-talk between tumor cells and immune cells have changed the direction of the understanding of molecular circuitry involved in OSCC [12]. Growing evidence suggest that immune cells including T lymphocytes, neutrophils, tumor-associated macrophages (TAMs), mast cells, as well as cytokines and cancer-associated fibroblasts (CAFs) are the critical inflammatory mediators of OSCC that promote or restrain oral carcinogenesis [4]. Innate immune cells are the first line of defenders in tumor microenvironment (TME) that not only do they eliminate tumor cells during early stage, but they also modulate TME to support tumor progression [13]. Amongst the new players in OSCC pathogenesis are ILCs [7]. In this study, we investigated lymphocyte infiltration, ILCs characterization, and cytokine expression in treatment naive OSCC patients and an in vivo model of OSCC. We first sought to assess lymphocytic infiltration in tumor tissues using the International Immuno-Oncology Biomarker Working Group (IBWG) guidelines, followed by in-depth analysis pertaining to ILCs in the OSCC environment [14,15].

Our data show significantly increased infiltration of lymphocyte in OSCC compared to non-tumor tissue, indicating that the immune response in OSCC is also previously described [16,17,18]. We further report that heavy lymphocytic infiltrate was a feature of the stroma-rich area and small sized tumors. These findings are in line with the existing body of literature that shows reduced lymphocytic infiltration in large tumors [14,16]. While the evaluation of lymphocytic infiltration in H & E-stained sections does not show different types of lymphocytes, it has been increasingly recognized as a prognostic biomarker for assessing tumor behavior in squamous cell carcinoma of the head and neck (SCCHN) [16]. Although we did not evaluate prognosis in our cohort of patients, previous studies have reported a better outcome with increased lymphocytic infiltration, including SCCHN, possibly due to antitumor immunity leading to the destruction of cancer cells and controlling the cancer progression [17,18].

ILCs are a heterogeneous population of immune cells that reside in tissues and have the propensity to expand under varying physiological and pathological states. Moreover, in response to cytokine production, ILC subsets have shown to exhibit plasticity in different cancers, including lung cancer and hepatocellular carcinoma [6,19,20]. Our results show that among the three ILC subsets in human OSCC, ILC3 infiltrate was most abundant, followed by ILC2 and ILC1. The literature regarding ILCs infiltration is highly dynamic. Many studies have reported that the frequencies of ILCs vary in different cancers as well as within the same cancer under different environmental conditions [21]. In line with previous evidence [21], our study shows variable frequencies of all three ILC subsets. We found an increased infiltration of ILC3 and ILC2 and a reduced frequency of ILC1 in OSCC. However, in contrast, a recent study showed enrichment of ILC1 and ILC3 and the complete absence of ILC2 in SCCHN [7]. The absence of the ILC2 subset could be due to the several differences between the two experimental settings. The first reason could be the risk factors. In Pakistan, chewed tobacco in the form of gutka, mawa, betel quid (pan), and naswar is frequently consumed, which is more carcinogenic than smoked tobacco, and the high concentration of carcinogenic ingredients and the frequency and duration of site-contact with chewed tobacco products prevail, causing inflammation and carcinogenesis [22,23,24]. In comparison, human papillomavirus (HPV) infection is a well-recognized factor, in addition to smoking and alcohol for SCCHN in Western countries. A study conducted in the Pakistani population with HPV-OSCC showed variation in driver genes mutation, including *FGFR2*, *ARID2, ALK*, *MLL3*, *MYC,* and *ASNS,* compared to dysregulated *FGFR3*, *E2F1*, and *TRAF3* in HPV+ tumors [25]. In the current study, we did not evaluate the HPV status in our samples; however, some samples in a previous study [7] showed positivity for p16 or HPV in situ hybridization, indicating that HPV status might lead to differences in immune cell profiles as well as mutational landscapes. However, in-depth studies need to be taken into account for ILCs in HPV+ and HPV− tumors. Another important reason could be the heterogeneity of ILC surface markers. To date, none of the markers are specific, and studies have utilized different markers for gating strategies that may result in slightly different ILC types under the same name, leading to increased data variability [26]. Among other human cancers, the frequency of ILC1 was high and ILC3 was low in gastric cancer tissues, whereas ILC2 was increased in the peripheral blood of gastric cancer patients [27]. Furthermore, the number of ILC3 was down in lung squamous cell carcinoma tissue and ILC2 was up in peripheral blood of lung cancer patients [28]. Intriguingly, in acute myeloid leukemia, ILC1 was increased and ILC3 was decreased at disease onset [29]. In breast cancer, ILC2s were enriched, whereas ILC1 and ILC3 remained the same [30]. Another study in breast cancer reported increased infiltration of ILC3 [31]. Taken together, the number of ILC subsets has diverse proportions in human cancers at disease onset, in tumor tissues versus blood, as well as within the same tumors in different studies, demanding frequent studies on this subject matter.

Our murine data show reduced ILC1 (the same as in human OSCC) and ILC3 infiltrate in OSCC compared to controls, while ILC2 infiltrate was not significantly changed. Importantly, we did observe ILCs in the acetone group as well, possibly due to inflammatory events in these mice. Nevertheless, ILC1 downregulation was significant in mice in the DMBA group, and this finding parallels our findings in human OSCC tissues. Previously, a study reported all ILC subsets in murine gingivae; however, the frequencies of ILC1 and ILC2 were relatively equal [32]. Furthermore, a study identified only ILC3 in the murine tongue, palate, and gingiva [33]. Recently, a study reported that ILC1s, not ILC3s, are the major patrolling cells in uninfected murine labial mucosa [34]. Taken together, the ILC compartments and their frequencies might differ due to site and function specificity, which could be further evaluated in future studies focusing on all ILC types in murine oral mucosal subsites [33].

In order to investigate the molecular circuitry involved in OSCC, we developed a murine OSCC model using DMBA followed by inhibition of ILCs using an anti-Thy1 antibody. We observed that, after anti-Thy1 antibody treatment in the DMBA+Ab group, the number of large tumors, tumor incidence, and lymphocyte infiltration were significantly reduced compared to the DMBA group, indicating that inhibiting ILCs limited tumor expansion and tumor burden. Furthermore, consistent with human OSCC, we found a concomitant decrease in ILC1 in the DMBA and DMBA+Ab groups compared to the acetone and control groups, indicating that mice with decreased ILC1 are more prone to developing chemically induced OSCC [35]. Similarly, an in vivo experiment showed a decreased frequency of ILC1 in the late stage of colorectal cancer [36]. Contrary to this, in the current study, an insignificant rise in ILC2s was found in the DMBA+Ab group compared to the DMBA, acetone, and control groups. In parallel, a previous study showed increased infiltration of ILC2 in murine colorectal carcinoma, and blocking ILC2 using antiCD90.2 in nude mice promoted tumor growth [36]. In the current study, ILC3 infiltration was higher in the DMBA+Ab treatment group compared to DMBA. To date, there is no data available to compare ILCs in the murine OSCC model. However, in contrast, mouse colorectal cancer showed a reduced frequency of ILC3 in the late stage of colorectal cancer [37].

Our data show upregulation of IFNγ in human OSCC. While we observed a low frequency of ILC1 in humans, it is possible that the aforementioned cytokine is secreted by sources other than ILC1 in the tumor niche. IFNγ, for example, is known to be secreted by T_H_ cells, cytotoxic T lymphocytes, and natural killer cells and has both a pro- and antitumor immune response in cancers. Increased levels of IFNγ have been shown to exert apoptosis and antitumor activity in SCCHN [5,38].

Expression of ILC2-associated cytokines (IL-2, IL-4, IL-6, and IL-10) was not different from controls. It is important to note here that we did not have a control group comparison as we did not find enough mononuclear cells in the controls. However, if we look at our murine data, the ILC2 infiltrate was not different in murine OSCC compared to controls. This may have an indirect explanation for the unchanged expression of ILC2-associated cytokines in human OSCC in our dataset. However, deregulated levels of IL-2, IL-4, IL-6, and IL-10 have been reported in SCCHN tissue or plasma [38].

Murine PCR data showed upregulation of IFNγ, IL-10, and IL-4. If we plot these findings alongside our ILC data, they do not match with reduced ILC1 and unchanged ILC2 expression. However, as stated for human PCR data alone, other sources for secretion of these cytokines cannot be ruled out [38].

We observed interesting patterns in the expression of IL17 and IL17a. IL-17 was high and IL-17a was low in both human and murine OSCC tissues compared to controls. Increased expression of IL-17 correlates with advanced tumor stage and metastasis in OSCC [38]. The Cancer Genomic Atlas database correlated decreased levels of IL-17a with advanced tumor stage in OSCC, whereas another study correlated high expression of IL-17a with tumor progression in tongue squamous cell carcinoma [39,40]. Furthermore, IL-17 (in the presence of IL-23) orchestrates the conversion of ILC1 into ILC3 and inhibits the adaptive arm of an immune response in lung squamous cell carcinoma and hepatocellular carcinoma [19,20]; however, this needs to be studied in OSCC. In contrast to our findings, higher levels of IFN and IL-17 were found in premalignant oral lesion tissues as well as regional lymph nodes compared to the levels in normal or OSCC tissues in both humans and mice [41]. The differences in our findings could be due to the methodological discrepancies between the studies and the complex tumor–immune interaction.

Like any other research study, we had a few limitations that are worth mentioning. Our murine model was immunocompetent, focusing only on ILCs. Secondly, CD90 (Thy1) is expressed on other immune cells, including T cells, fibroblasts, basophils, as well as dendritic and endothelial cells [42,43], and thus the anti-Thy1 antibody might have inhibited pro-tumor cells, resulting in decelerated tumorigenesis and limited tumor expansion and a non-significant difference in ILCs in the DMBA and DMBA+Ab groups. However, this warrants further investigation via CD90 staining along with other panels, which will enable us not only to delineate specific cellular categories but also to localize their infiltration. However, it is important to mention that due to a lot of “gating-out” prerequisites for ILCs identification, IHC has not been recommended to date to investigate ILCs infiltration. Finally, we were unable to investigate ILCs subsets (ILC1, ILC2, and ILC3) in segregated murine tumor categories (small, intermediate, and large) primarily because (a) most mice had a combination of various sized tumors and (b) because of a lot of “gating-out” strategies to identify ILCs on flow cytometer, a small population would have been achieved if segregated tumors were approached to isolate ILCs. However, we recommend pooling-in small, intermediate, and large tumors from various mice to look into which ILC category is more abundant/reduced in each tumor size category.

Together, we report a murine model of OSCC in NMRI outbred mice. The anti-Thy1 antibody reduced tumor burden and limited tumor expansion in DMBA+Ab mice and did not have any effect on ILCs. We also report reduced populations of ILC1 in human and murine OSCC tissues and upregulation of IFNγ as well as IL17 and downregulation of IL-17a in human and murine OSCC tissues. Future studies focusing on ILCs as well as intracellular cytokines staining in OSCC in Rag-/- and T cell deficient/knock-out mice are suggested.

## 4. Materials and Methods

### 4.1. Patients

In this study, a total of 52 consecutive patients with biopsy-proven primary oral squamous cell carcinoma (OSCC) and 6 non-tumor adjacent tissue controls from patients who underwent surgical resection of tumors were recruited. This study was approved by the ethical review board (Ref#IRB-1097/DUHS/Approval/2018/104) of the Dow University of Health Sciences (DUHS), and informed consent was obtained before patient recruitment. Demographic and histopathological details, including age, gender, history of tobacco addiction, tumor site, size, grade, pathological stage of the tumor, perineural invasion, lymphovascular invasion, and lymph node metastasis, were recorded in performa. Patients with chemoradiotherapy, recurrent oral squamous cell carcinoma, a history of any other cancer in body and systemic diseases, as well as the involvement of tumors in normal tissues adjacent to tumors, were excluded from study.

### 4.2. Murine Experiments

In order to investigate the functional characterization of ILCs in OSCC, we induced murine OSCC in the Naval Medical Research Institute (NMRI)-outbred-strain, as previously published [44]. Briefly, 73 immunocompetent, healthy male NMRI outbred strain, 20 ± 2 gm, and 6–8 weeks old at the time of recruitment were obtained from the animal house of DUHS. Mice were randomly divided into 4 groups: 7,12-dimethylbenz[a]anthracene (the DMBA group, n = 33), the DMBA+antibody (Ab) group (n = 30), the acetone group (n = 5), and the control group (n = 5). All mice were housed in conventional propylene plastic cages and covered with a filter top, with five mice in each cage. All experiments were run in a 12-h light and dark ratio, ±26 °C temperature, ±55% humidity with normal chow feed, and water ad libitum. After a week of acclimatization, mice in the DMBA and DMBA+Ab groups were topically brushed twice with 0.5% DMBA solution (0.025 g DMBA dissolved in 5 mL acetone) on the lower lip at every alternate day for 20 weeks. In order to block ILCs, mice in the DMBA+Ab group were injected with 300 µL of α-Thy1 antibody (30H12Clone) twice weekly intraperitoneally. Mice in the acetone group were brushed with acetone on the lower lip every alternate day for 20 weeks and the control group was given nothing. Mice were observed daily for illnesses and weighed weekly. All animals were culled at week 20 through cervical dislocation, following the Animals (Scientific Procedures) Act guidelines, and harvested tissues were analyzed for histopathological diagnosis, ILCs subsets, and cytokines. All murine experiments were performed at the animal testing facility, the Dow Institute for Advanced Biological and Animal Research. The study was approved by the university review board (Ref# AR.IRB-013/DUHS/Approval/2018/020) of DUHS for animal research and followed ARRIVE guidelines.

### 4.3. Quantification of Lymphocyte Infiltration in Human and Murine OSCC

Initially, lymphocyte infiltration was evaluated in human and murine OSCC tissues using hematoxylin and eosin (H & E) whole slides as per the International Immuno-Oncology Biomarker Working Group (IBWG) guidelines [14,15]. Lymphocytes were assessed in whole slide and OSCC samples under investigation were stratified as either (a) tumor cell-rich, (b) stroma-rich, or (c) moderate. The H & E slides were scanned at low magnification (x100 and x200 for murine and human, respectively), followed by higher magnification at x400 under a compound microscope (Nikon eclipse-80i Japan). Lymphocytes were evaluated by two investigators who were blinded to the clinicopathological features of the patients.

### 4.4. Isolation of Mononuclear Cells

Excised tumor tissues were rinsed with phosphate buffered saline (PBS) and minced into small pieces via sharp scissors. Following mincing, tumor tissues were transported to a 50 mL falcon tube containing 25 mL of digest solution (0.0125 gm collagenase (Gibco, USA), 2.5 mL fetal-bovine serum (Gibco), 0.5 mL penicillin-streptomycin (Gibco, USA), 0.5 mL L-glutamine (HyClone, USA), and 46.5 mL Roswell Park Institute Medium (Gibco, USA)) and incubated at 37 °C for 45 min. The solution was then filtered via a 100 µm strainer (93100, SPL life sciences, Korea) in a fresh 50 mL falcon tube. Following this, 10 mL ficoll-paque density gradient (Cytiva, Sweden) was poured into a fresh 50 mL falcon tube and the filtered solution was steadily poured alongside the wall of the falcon tube via a plastic pasteur pipette and centrifuged at 1000× *g* for 20 min at 20 °C with 3 accelerations and no break. The middle layer containing mononuclear cells were isolated in 15 mL falcon tubes and centrifuged at 10,000 rpm for 10 min at 20 °C. The supernatant was discarded, and the cell pellet was resuspended in 1 mL PBS and re-centrifuged. The final pellet was resuspended in 1200 µL cold PBS. Subsequently, 300 µL of cell solution were transported to 4 Eppendorf tubes labeled as ILC1, ILC2, ILC3, and control. Fc block (1 µL) was added in all tubes except in control and allowed to incubate in dark for 15 min at room temperature. Subsequently, 0.5 µg of each primary labeled antibody was added for staining ILC1, ILC2, and ILC3 followed by incubation at 4 °C for 30 min, centrifugation at 1500 rpm for 7 min, and resuspension of the cell pellet in 500 µL PBS. The samples were introduced to a BD FACS Canto flow cytometer. The dead cells were excluded using 7-AAD viable dye.

### 4.5. ILCs Identification via Flowcytometry

Human ILCs were identified using the following gating strategy. ILC1: CD45+ (eBioscience 11045941), LIN-Eomes- (eBioscience 46487741); ILC2: CD45+ LIN- CD90+; and ILC3: CD45+ LIN- RORγt+ (eBioscience 12698880). For gating in/out of LIN- cells, CD3+ (eBioscience 11003741), CD8+ CD19+ were gated outed. Murine ILCs were identified as: ILC1: CD45+ (BD Pharmingen 557659), LIN- CD335- (BD Pharmingen 560755, 560757), CD49b- (BD Pharmingen 50628); ILC2: CD45+ LIN- CD90.2+ (BD Pharmingen 553014); and ILC3: CD45+ LIN- RORγt+ (BD Pharmingen 562607). For gating in/out of LIN- cells, CD3+ (BD Pharmingen 553061), CD19+ (BD Pharmingen 557398), and CD4+ (BD Pharmingen 553046) were gated out. Analyses were performed using FlowJo software v10.8.1. ILCs percentages were mentioned as the percentage of CD45+ LIN- cells.

### 4.6. RNA Extraction from Murine and Human OSCC Tissues

Total RNA was extracted from fresh frozen tissue using Trizol reagent. Briefly, tissue homogenization was performed at 6000 rpm, 3 × 15 s and 5 s interval in a homogenizer (Percelly’s 24 Bertin, France) with ceramic beads (2.8 mm size) and 1 mL Trizol reagent (Ambion, life technologies, USA). Following homogenization, phase separation was carried out using 200 µL chloroform and centrifugation 12,000 rpm at 4 °C for 15 min. The aqueous phase containing RNA was precipitated by the addition of isopropanol (500 µL) and centrifuged at 12,000 rpm at 4 °C for 15 min. The RNA-pellet was washed with 70% ethanol (1 mL), centrifuged at 7500 rpm at 4 °C for 5 min, and diluted in RNAse-free water (20 µL). Pure RNA between 1.8–2.2 at A_260_/A_280_ ratio was considered for further processing.

### 4.7. Complementary DNA (cDNA) Synthesis

Complementary DNA (cDNA) synthesis was performed according to the manufacturer’s guidelines, using a cDNA synthesis kit (cat# K1622, Themoscientific, USA). Briefly, in a 20 µL reaction mix, RNA (5 µL), random hexamer primer (1 µL), reaction-buffer (4 µL 5X), 20 U/µL riboLock RNAse-inhibitor (1 µL), 10 mm dNTP (2 µL) and 200 U/µL RevertAid M-MuLV RT (1 µL), and nuclease free water (6 µL) were added. Single-cycling was performed using a thermal cycler (Bio-Rad, Singapore) with annealing (25 °C, 5 min), reverse transcription (42 °C, 60 min), and extension (70 °C, 5 min).

### 4.8. Quantitative Real Time Polymerase Chain Reaction (RT-qPCR)

A quantitative real-time polymerase chain reaction (RT-qPCR) was performed using a SYBR green Maxima kit (K0221, Thermoscientific) as per the manufacturer’s guidelines. In a total of 25 µL master mix, 12.5 µL SYBR green, 1 µL primers (forward and reverse), 10.5 µL water, and 1 µL cDNA were added. The reactions were performed in duplicates in 72-well plates Rotor-Gene (Qiagen) with the initial denaturation at 95 °C for 10 min, 45 cycles at 95 °C for 15 s, and annealing at 60 °C for 1 min. The samples were acquired to melting for the specificity of amplicons. The samples were normalized with glyceraldehyde-3-phosphate dehydrogenase (GAPDH) and forward and reverse primers (sequence 5′–3′) were used from previous studies [45,46,47,48,49,50] (Table 2 and Table 3).

## Figures and Tables

**Table 1:** Clinicopathologic characteristics of oral squamous cell carcinoma (OSCC) patients.

Characteristics (N = 52)	Frequency (%)
**Mean Age in years**	
Male	46
Female	57.4
**Gender**	
Male	42 (80.8)
Female	10 (19.2)
**Site**	
Buccal mucosa	28 (53.8)
Tongue	10 (19.2)
Lip	05 (9.7)
Alveolus	05 (9.7)
Hard palate	04 (7.6)
**Tobacco Habits**	
Yes	42 (80.8)
No	10 (19.2)
**Type of biopsy**	
Surgical excision	29 (55.8)
Punch/incisional	23 (44.2)
**Grade**	
Moderately differentiated	38 (73.1)
Well differentiated	09 (17.3)
Poorly differentiated	05 (9.6)
Size * (n = 29)	
pT1	02 (7.0)
pT2	13 (44.8)
pT3	03 (10.3)
pT4	11 (37.9)
Nodal metastasis * (n = 29)	
pN0	15 (51.7)
pN1	03 (10.3)
pN2	06 (20.8)
pN3	05 (17.2)
**Lymphovascular invasion (n = 29)**	
No	27 (93.1)
Yes	02 (6.9)
**Perineural invasion (n = 29)**	
No	18 (62.1)
Yes	11 (37.9)

*p*—pathological; T—tumor; N—node; * pTNM staging as per College of American Pathologist (CAP) protocol.

**Table 2:** Primer sequence of the target genes of human oral squamous cell carcinoma tissues.

Human Cytokines	Primer Sequence (5′–3′)	Length
IL-2 [45]	F: AACTCACCAGGATGCTCACATTTA R: TCCCTGGGTCTTAAGTGAAAGTTT	24 24
IL-4 [45]	F: CCACGGACACAAGTGCGATA R: CCCTGCAGAAGGTTTCCTTCT	20 21
IL-6 [46]	F: CCACTCACCTCTTCAGAACGAAT R: TTGGAAGCATCCATCTTTTTCA	23 22
IL-10 [45]	F: GTGATGCCCCAAGCTGAGA R: CACGGCCTTGCTCTTGTTTT	19 20
IL-17 [47]	F: TCAACCCGATTGTCCACCAT R: GAGTTTAGTCCGAAATGAGGCTG	20 23
IL-17a [48]	F: CAT CCA TAA CCG GAA TAC CAA TA R: TAG TCC ACG TTC CCA TCA GC	23 20
IFN-γ [45]	F: TCAGCTCTGCATCGTTTTGG R: GTTCCATTATCCGCTACATCTGAA	20 24
GAPDH	F: GCATCCTGGGCTACACTGA R: CCACCACCCTGTTGCTGTA	19 19

IL—interleukin; IFN-γ—Interferon gamma; F—forward; and R—Reverse.

**Table 3:** Primer sequence of the target genes of murine oral squamous cell carcinoma tissues.

Murine Cytokines	Primer Sequence (5′–3′)	Length
IL-2 [45]	F: CCTGAGCAGGATGGAGAATTACA R: TCCAGAACATGCCGCAGAG	23 19
IL-4 [45]	F: ACAGGAGAAGGGACGCCAT R: GAAGCCCTACAGACGAGCTCA	19 21
IL-6 [45]	F: GAGGATACCACTCCCAACAGACC R: AAGTGCATCATCGTTGTTCATACA	23 24
IL-10 [45]	F: GGTTGCCAAGCCTTATCGGA R: ACCTGCTCCACTGCCTTGCT	20 20
IL-17 [45]	F: GCTCCAGAAGGCCCTCAGA R: AGCTTTCCCTCCGCATTGA	19 19
IL-17a [49]	F: ATCCCTCAAAGCTCAGCGTGTC R: GGGTCTTCATTGCGGTGGAGAG	22 22
IFN-γ [45]	F: TCAAGTGGCATAGATGTGGAAGAA R: TGGCTCTGCAGGATTTTCATG	24 21
GAPDH [50]	F: TTCACCACCATGGAGAAGGC R: GGCATGGACTGTGGTCATGA	20 20

## Data Availability

Not applicable.

## Supplementary Materials

The following supporting information can be downloaded at: https://www.mdpi.com/article/10.3390/ijms24021627/s1.
