# Supplementary material for: Differential Regulation of Innate Lymphoid Cells in Human and Murine Oral Squamous Cell Carcinoma
Source: Int J Mol Sci. 2023 Jan 13;24(2):1627. doi: 10.3390/ijms24021627 (PMC9865302; doi:10.3390/ijms24021627)
Supplement: Supplementary file 1 [file ijms-24-01627-s001.zip › ijms-2105496-supplementary.pdf]

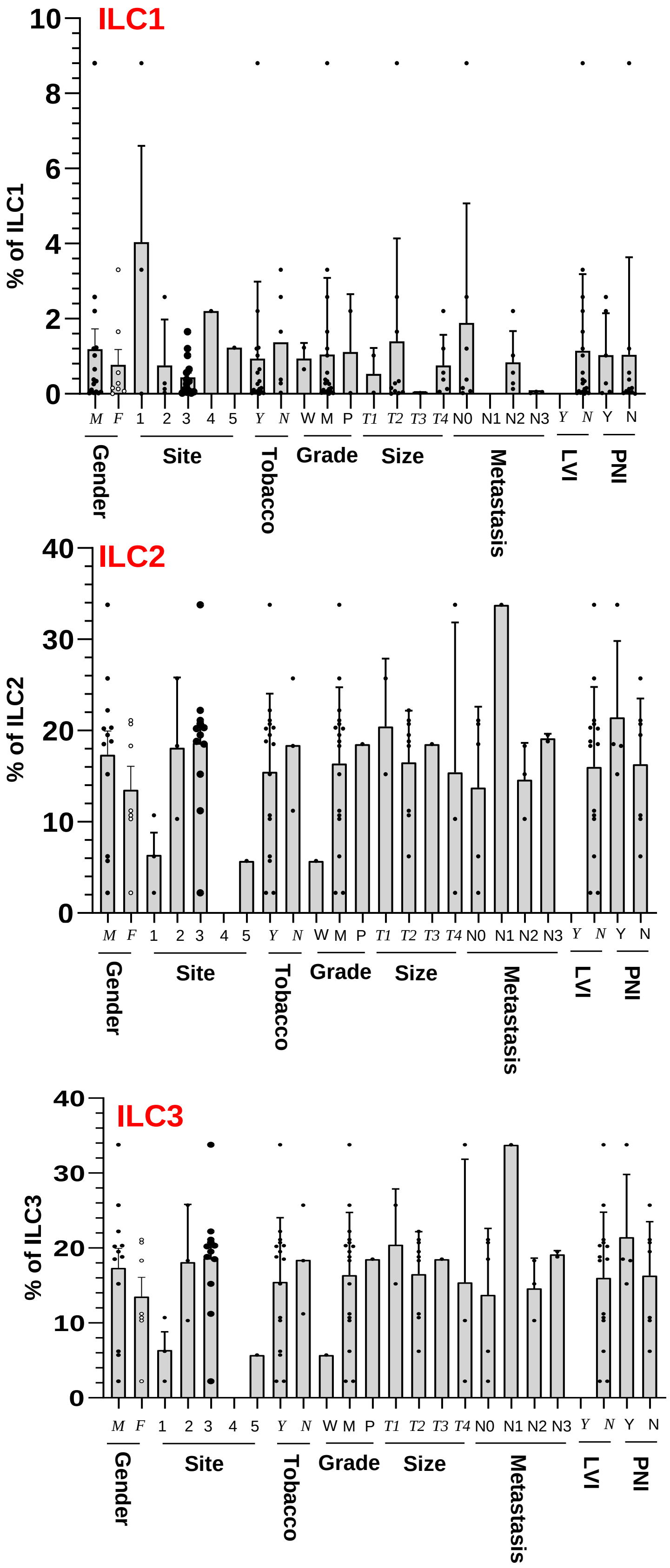

ILC1
% of ILC1
ILC2
% of ILC2
ILC3
% of ILC3
M F 1 2 3 4 5 Y N W M P T1 T2 T3 T4 N0 N1 N2 N3 Y N Y N
Gender
Site
Tobacco
Grade
Size
Metastasis
LVI
PNI

Supplementary Figure S1: ILCs infiltration segregated across various clinicopathological parameters in OSCC patients

Percentages of ILC1, ILC2 and ILC3 are plotted separately against each of the category in various clinicopathological parameters in OSCC patients. X-axis denotes various clinicopathological parameters and Y-axis denotes percentages of ILCs.

M=male, F=female, 1=buccal mucosa, 2=lip, 3=tongue, 4=alveolus, 5=hard palate, Y=yes, N=no, W=well differentiated, M=moderately differentiated, P=poorly differentiate, T1=<2cm, T2=2-4cm, T3=>4cm, T4=>4cm+locally advanced, N0=no lymph node metastatis, N1=single ipsilateral lymph node involved at ≤3cm, N2=single/multiple ipsilateral, bilateral/contralateral lymph node at 3-6cm, N3= metastasis in lymph node at >6cm in its greatest dimension.
